# High‐Energy (>10 MeV) Oxygen and Sulfur Ions Observed at Jupiter From Pulse Width Measurements of the JEDI Sensors

**DOI:** 10.1029/2019GL083842

**Published:** 2019-10-28

**Authors:** J. H. Westlake, G. Clark, D. K. Haggerty, S. E. Jaskulek, P. Kollmann, B. H. Mauk, D. G. Mitchell, K. S. Nelson, C. P. Paranicas, A. M. Rymer

**Affiliations:** ^1^ Johns Hopkins University Applied Physics Laboratory Laurel MD USA

**Keywords:** Jupiter, aurora, X‐ray, Io, energetic particles, Juno

## Abstract

The Jovian polar regions produce X‐rays that are characteristic of very energetic oxygen and sulfur that become highly charged on precipitating into Jupiter's upper atmosphere. Juno has traversed the polar regions above where these energetic ions are expected to be precipitating revealing a complex composition and energy structure. Energetic ions are likely to drive the characteristic X‐rays observed at Jupiter (Haggerty et al., 2017, https://doi.org/10.1002/2017GL072866; Houston et al., 2018, https://doi.org/10.1002/2017JA024872; Kharchenko et al., 2006, https://doi.org/10.1029/2006GL026039). Motivated by the science of X‐ray generation, we describe here Juno Jupiter Energetic Particle Detector Instrument (JEDI) measurements of ions above 1 MeV and demonstrate the capability of measuring oxygen and sulfur ions with energies up to 100 MeV. We detail the process of retrieving ion fluxes from pulse width data on instruments like JEDI (called “puck's”; Clark, Cohen, et al., 2016, https://doi.org/10.1002/2017GL074366; Clark, Mauk, et al., 2016, https://doi.org/10.1002/2015JA022257; Mauk et al., 2013, https://doi.org/10.1007/s11214-013-0025-3) as well as details on retrieving very energetic particles (>20 MeV) above which the pulse width also saturates.

## Key Points

- The Juno JEDI instrument is shown to have the unplanned capability to measure heavy ions to energies as high as 100 MeV
- As such, the JEDI instrument has the capability to measure those ions needed to generate polar X‐rays at Jupiter (greater than tens of megaelectron volts O and/or S)
- Although not yet directly correlated with polar X‐rays, we show that heavy ions up to 100 MeV are indeed observed over Jupiter's polar regions

## 1 Introduction

Jupiter has two distinct regions of X‐ray emissions: the low‐latitude region and the high‐latitude, or auroral, region. The regions are treated distinctly because it is thought that they are produced by different physical mechanisms. The low‐latitude X‐ray emissions are generated via scattering of solar X‐rays in Jupiter's atmosphere, whereas the high‐latitude X‐rays are generated via bremsstrahlung and charge stripping of oxygen and sulfur ions. The later mechanism is thought to be the dominant one. (Bhardwaj et al., 2005; Branduardi‐Raymont et al., 2008; Cravens et al., 1995; Dunn et al., 2017; Gladstone et al., 2002; Horanyi et al., 1988; Maurellis et al., 2000; Ozak et al., 2010).

The characteristic emissions from oxygen and sulfur are produced when energetic ions precipitate into the upper atmosphere, become highly charged by collisions with atmospheric constituents, and produce soft X‐ray photons from transitions between high‐energy electronic atomic orbital states (e.g., Houston et al., 2018). These characteristic emissions are thought to be associated with polar region hot spots that are distinct from, and poleward of, the main oval emission dominated by bremsstrahlung.

The source of the energetic ions to power the polar X‐ray emissions has always been assumed to be the sulfur and oxygen ions that are transported from the Io plasma torus to permeate the more distant regions of the magnetosphere that connect magnetically to the X‐ray emission regions. The magnetospheric system energizes some of these heavy ions to megaelectron volt energies as they are transported outward. At low altitudes over Jupiter's poles, Clark et al. (2017) discovered regions mapping to Jupiter's middle magnetosphere with large parallel potentials (tens to hundreds of kilovolts) capable of accelerating ions downward into the atmosphere. Additionally, Clark et al. (2017) found that oxygen and sulfur were accelerated to similar energies in the DC potential; thus, the ions were likely similar charge states and magnetospheric in origin (e.g., Clark, Mauk, et al., 2016). However, these ions require much higher energies (>0.5 to 1 MeV per nucleon or >8–16 MeV for oxygen and 16–32 MeV for sulfur) as they stream into the Jovian upper atmosphere to produce the highly stripped states needed to produce the observed X‐ray line emission; lower energies would stop within the atmosphere without yielding the high charge states required for characteristic line emission (Houston et al., 2018). While heavy ions are observed within the middle magnetosphere with sufficient energy (Mauk et al., 2004), the intensity of those observed ions appears to be far below those needed to account for the X‐ray emission intensities. Thus, additional mechanisms of acceleration along the connecting field lines have been invoked to achieve closure between the ion measurements and the X‐ray emission intensities.

Haggerty et al. (2017) reported first the direct observation of precipitating energetic heavy ions for a single pass over the polar regions. These observations showed significant fluxes of oxygen and sulfur ions above 1 MeV (up to 10 MeV) as well as the identification of additional ion species, potentially magnesium or sodium. However, the observed ions had insufficient flux (according to models) to drive the polar X‐ray emissions, and the authors pointed to the limited spatial and temporal extent of the observed X‐ray hot spots as a possible explanation. The Juno mission to date comprises over 30 combined passes of the northern and southern polar regions. Additionally, while the reported measurements had energies that were very high, the energies were still below those anticipated to be needed to generate the most intense X‐ray emissions.

The present work reports observations of ions with energies greater than 10 MeV precipitating into Jupiter's auroral zone. These ions are thought to be the ones with high enough velocity to cause them to be sufficiently stripped to generate the X‐rays. Specifically, early theoretical work suggested that energies >0.5 MeV per nucleon were needed (>8‐MeV oxygen and >16 MeV for sulfur; Cravens et al., 1995). More recent work suggests that >1.0 MeV per nucleon may be needed (>16‐MeV oxygen and >32 MeV for sulfur; Houston et al., 2018). Ions with such energies were discovered within the raw data stream of the Juno JEDI sensor that is produced sporadically as a data quality check. We use these observations to correlate these emissions with auroral features. The observations point to oxygen and sulfur ions with energies up to 100 MeV produced within the Jovian magnetosphere.

## 2 Materials and Methods

The Juno JEDI instrument (Mauk et al., 2013) is based on the Johns Hopkins University Applied Physics Laboratory's cylindrical energetic particle telescope design referred to as the “hockey puck” design (Clark, Cohen, et al., 2016; Clark, Mauk, et al., 2016). Energetic ions that penetrate the entrance foil of JEDI will then traverse the time‐of flight section, which uses signals produced by electrons that are created when an energetic ion traverses thin carbon foils to measure the time of flight (TOF) of these ions. The ions then impact a solid‐state detector (SSD) where they deposit their energy, and the resultant pulse height and pulse width is recorded. The two methods of extracting the SSD energy deposition greatly expand the dynamic range of the energy measurement. With the pulse height technique only, the energy measurements are limited to about 20 to 1,500 keV. With the combined techniques, we extend the range from 20 keV to 10 MeV as has been described by Haggerty et al. (2017). Here we discuss how the JEDI data are used to expand that range up to 100 MeV.

**Figure 1:**
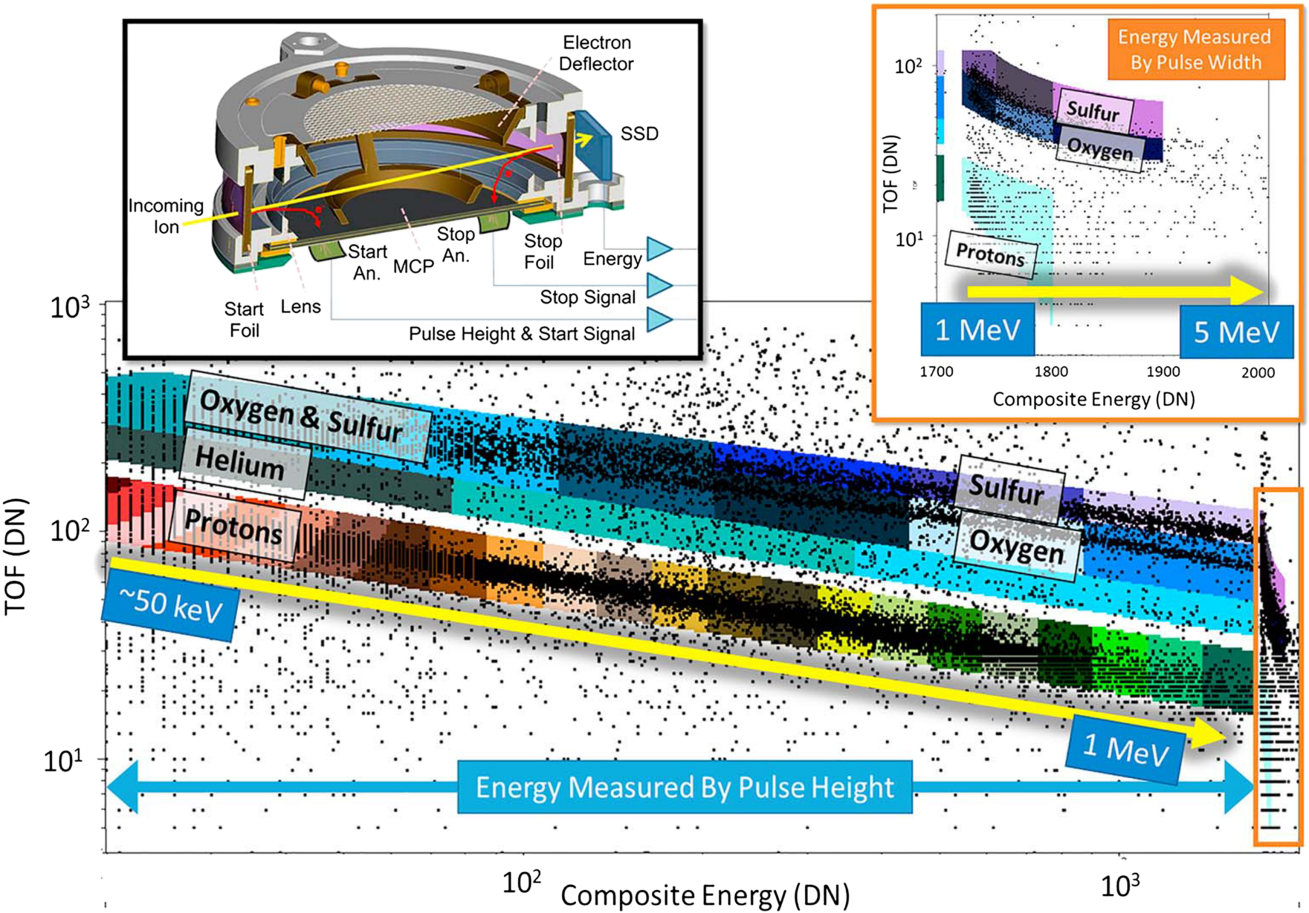
The upper left panel shows a cutaway of the Juno JEDI sensor following the path of an ion through the instrument where the energy is measured by the SSD, and the TOF is measured by the microchannel plate (MCP). The lower panel shows the raw TOF versus energy (TOFxE) data in data numbers (DN; see below for conversions to physical units) from Juno JEDI during the time period from 09:00 to 16:00 on 2018 day 38. The data show clearly separated tracks for protons, helium, oxygen, and sulfur. In the lower plot the energy is measured using the SSD pulse height. The points are the raw data points, and the colored boxes show the locations of the channel bins used to bin the data onboard. The inset in the upper right shows the TOF versus energy data measured using the pulse width for particles that have energies above about 1–2 MeV. SSD = solid‐state detector; TOF = time of flight.

The major fraction of the JEDI data that is sent to the ground is “channel” data. Within this channel data, the instrument measures the particle TOF as it traverses the 6‐cm flight path and the energy deposited onto a SSD. The instrument then places this measurement of TOF and energy (TOFxE) into a bin to be counted along with similar events. Figure 1 shows an example of the TOFxE measurement (black points) along with the predefined bins (colored boxes). The bin locations have been defined in TOFxE space using ground and in‐flight calibrations and have been adjusted during the mission based on raw data. The binned data allow for all TOFxE events to be characterized by species and energy and counted within this reduced data set. The binned data are accumulated over some number of seconds and are then telemetered and generally used as the main data products from the Juno JEDI instrument.

When possible, the instrument also sends down raw data that contain single events. As opposed to the channel data which contains a count of events that occurs within a range of TOF and energy values, the raw data contain the exact TOF and energy value for a single event. This data can therefore better resolve the exact TOF and *E* of the observed particles but not give a statistical count or flux of the particles. In this study we focus on the raw data products produced by the Juno JEDI instrument to find a class of particles that do not fall into one of the channel data products and represent oxygen and sulfur ions with energies up to 100 MeV.

## 3 Observations

Figure 1 shows a raw data plot that is characteristic in TOFxE measurements of a multispecies particle distribution at Jupiter. The raw data points align within “tracks” that have characteristic shapes which, given other factors like energy losses and inefficiencies, roughly trace the relation between the kinetic energy of the particle and its velocity of ***E***^**2**^ ***=*** (***pc***)^**2**^ ***+*** (***mc***^**2**^)^**2**^ where the relativistic correction can be largely ignored here resulting in a simplified E=12mv2. Each of these tracks is produced by different species within the particle population. In the Jovian magnetosphere and polar region these tracks correspond primarily to hydrogen, helium, oxygen, and sulfur, with hydrogen being the track with the lowest TOF per energy deposited and sulfur having the largest TOF per energy deposited. Note that JEDI does not measure charge state of the ions; the first foil encountered by the incoming particle completely rearrange the charge states of the ions (e.g., Allegrini et al., 2014), and so the charge state of the primary ion is not preserved inside the sensor in any case.

**Figure 2:**
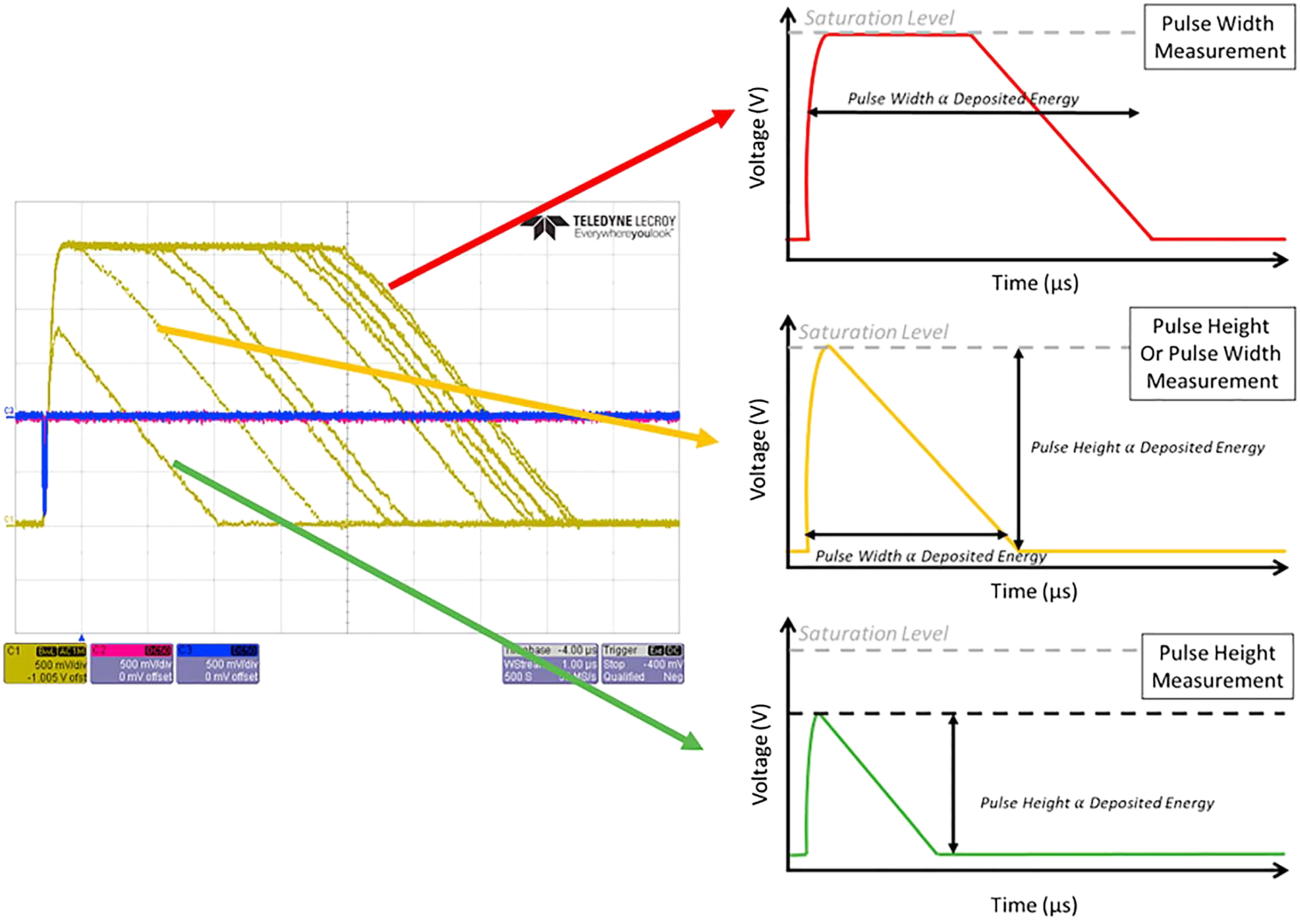
The figure on the left contains raw oscilloscope traces produced from a degraded Am‐241 alpha source on a solid‐state detector using the same energy chips used on Juno JEDI demonstrating their response to various energies of particles. The yellow traces are the output of the energy board analog output, while the blue and red traces are the start and stop anode pulses from a similar time of flight system on the Solar Probe EPI‐Lo instrument (Hill et al., 2017). The three figures on the right are a schematic of the measurement technique showing unsaturated pulse height measurements (green; under ~1–2 MeV), pulses nearly at saturation levels (yellow; ~2 MeV), and saturated pulse height measurements requiring a pulse width measurement (red; above ~2 MeV).

The JEDI instrument measures the ion energy using pulses produced by SSD, in this case 500‐μm‐thick silicon detectors. Raw pulses from a typical SSD, as amplified and shaped in an Application Specific Integrated Circuit (ASIC) used in Johns Hopkins University Applied Physics Laboratory particle instruments, are shown in Figure 2. For particles below about 2‐MeV deposited energy, the pulse shaping circuitry used in the ASIC remains completely linear (unsaturated; green line in Figure 2). For particles above about 2 MeV, the front‐end preamplifier and shaper circuitry remains in their linear range, but the output stage saturates, so the pulse height stops increasing with increased input energies (red line in Figure 2). The amount of time it takes for the output stage to slowly ramp down is related to how hard the output stage was driven into saturation and is therefore linearly related to the input energy. By measuring the time that the signal is over a threshold voltage, we can attain a measurement of the pulse width functionally extending the ASICs response in energy until the front‐end amplifier saturates (up to ~25 MeV).

**Figure 3:**
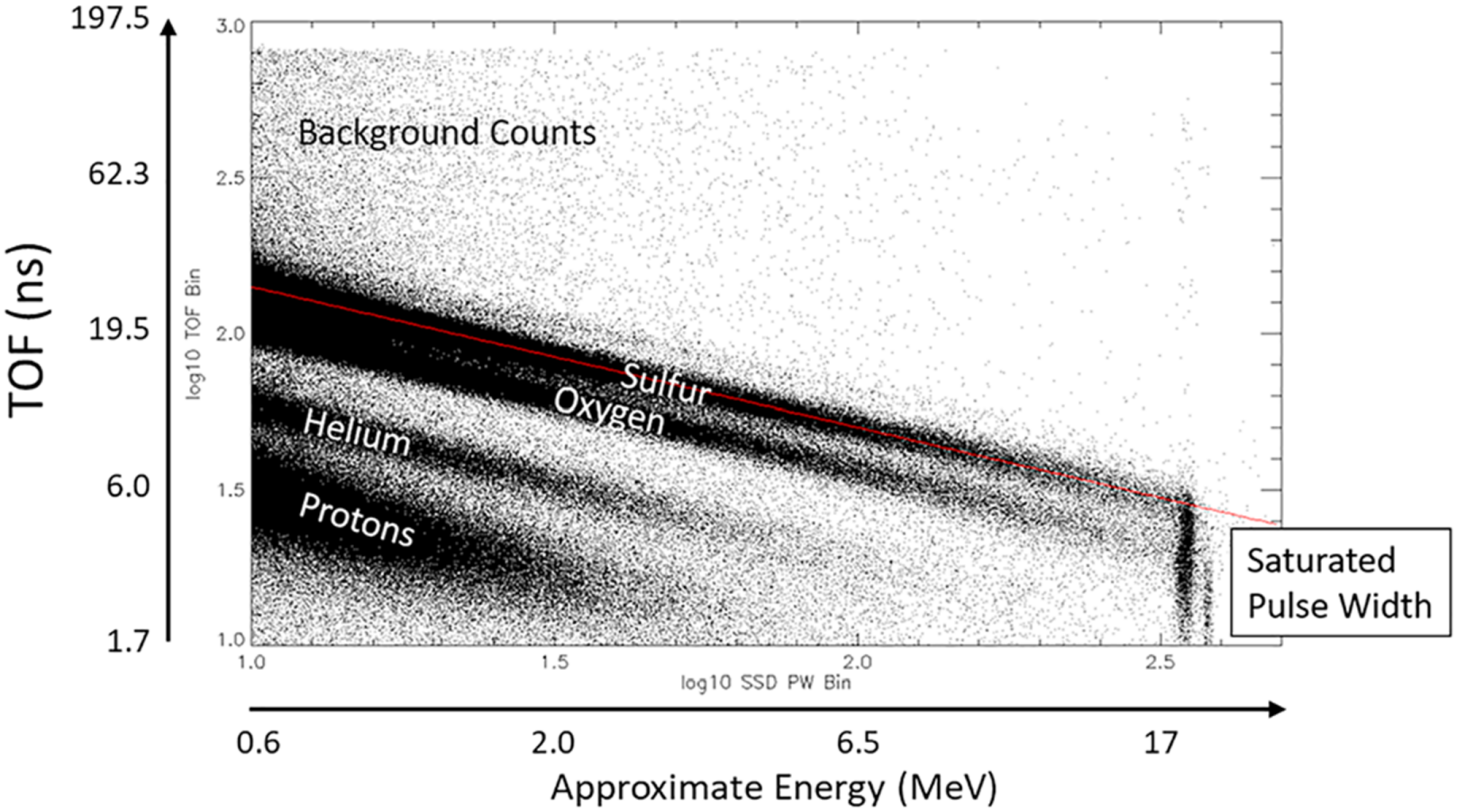
Particle time of flight versus pulse width measurements from 1 January 2006 to 30 October 2018 from the Juno JEDI‐90 sensor. These data represent several orbits in the Jupiter system and are a compilation of data retrieved from many locations within the magnetosphere. The secondary *y* axis gives the calibrated TOF through the 6‐cm path length, and the secondary *x* axis gives the approximate energy of the particles. The proton, helium, oxygen, and sulfur tracks are clearly identifiable against the background counts. The vertical clusters to the right of this plot are locations where the pulse width has saturated but still produced a unique TOF measurement. There are also some minor tracks visible within this figure, including one that appears to an ion heavier than sulfur. Within the TOFxPH data, Haggerty et al. (2017) noted an additional track between the oxygen and sulfur tracks likely associated with magnesium or sodium as well as a peak above the sulfur that could be associated with potassium. TOF = time of flight.

Although the pulse width response of the ASIC saturates around 25 MeV, the sensor energy range can be extended further by using TOF alone. The saturated pulse width measurements are seen in Figure 3 as the population of points that align in a vertical line to the right of the plot. These measurements have saturated pulse heights and saturated pulse widths but still have good TOF measurements. However, with only the TOF measurement, which measures only the energy/mass ratio, makes it challenging to determine particle composition. We do, howeverm have two additional pieces of information to bring to bear in this problem: (1) the composition must be consistent with the rest of the TOFxE tracks and (2) light ions (H or He) with TOF values this small would have enough energy to completely penetrate the SSD leaving only the minimum ionizing energy. Therefore, we know that these particles are heavy ions (S or O) and not light ions (H or He) and for the purposes of understanding X‐ray emission intensities, simply knowing that the ion is O or S tells us a great deal of what we need to know. We also note that this region is generally devoid of background counts as the probability to get a very large, very short pulse in the instrument that is not associated with one of these ions is very small.

To determine the energy of these heavy ions, we use the calculated TOF through the 6‐cm path length. We convert from the data number to TOF using the preflight calibrated value of TOF (ns) = TOF (DN)*0.1978 − 0.2598. Then the energy is given in electron volts by

$$E\;\left(\mathrm{eV}\right)=\frac{1}{2}mv^{2}=\frac{1}{2}m{\left(\frac{0.06}{10^{-9}\left(\mathrm{TOF}\left(\mathrm{DN}\right)*0.1978-0.2598\right)}\right)}^{2}$$

The observations shown in Figure 3 therefore show protons and helium with energies up to a few megaelectron volts and oxygen and sulfur with energies up to possibly hundreds of megaelectron volts.

Figure 3 shows the raw TOFxPW data on a log‐log plot obtained by JEDI‐90 over the time period from 1 January 2016 to 30 October 2018 and includes over 20 million measurements. The proton, helium, oxygen, and sulfur tracks are labeled in the figure, easily identifiable in the data, and consistent with the lower‐energy TOFxPH measurements. The secondary *x* and *y* axes show values of the TOF and the approximate energy for the ions based on their TOF alone. The vertical line to the right of the plot is the saturated pulse width measurements that extend from a TOF that ranges from 1.7 to 5.3 ns. Assuming that the composition is oxygen and sulfur, this corresponds to an energy range of 22–101 MeV for oxygen and 21–202 MeV for sulfur.

**Figure 4:**
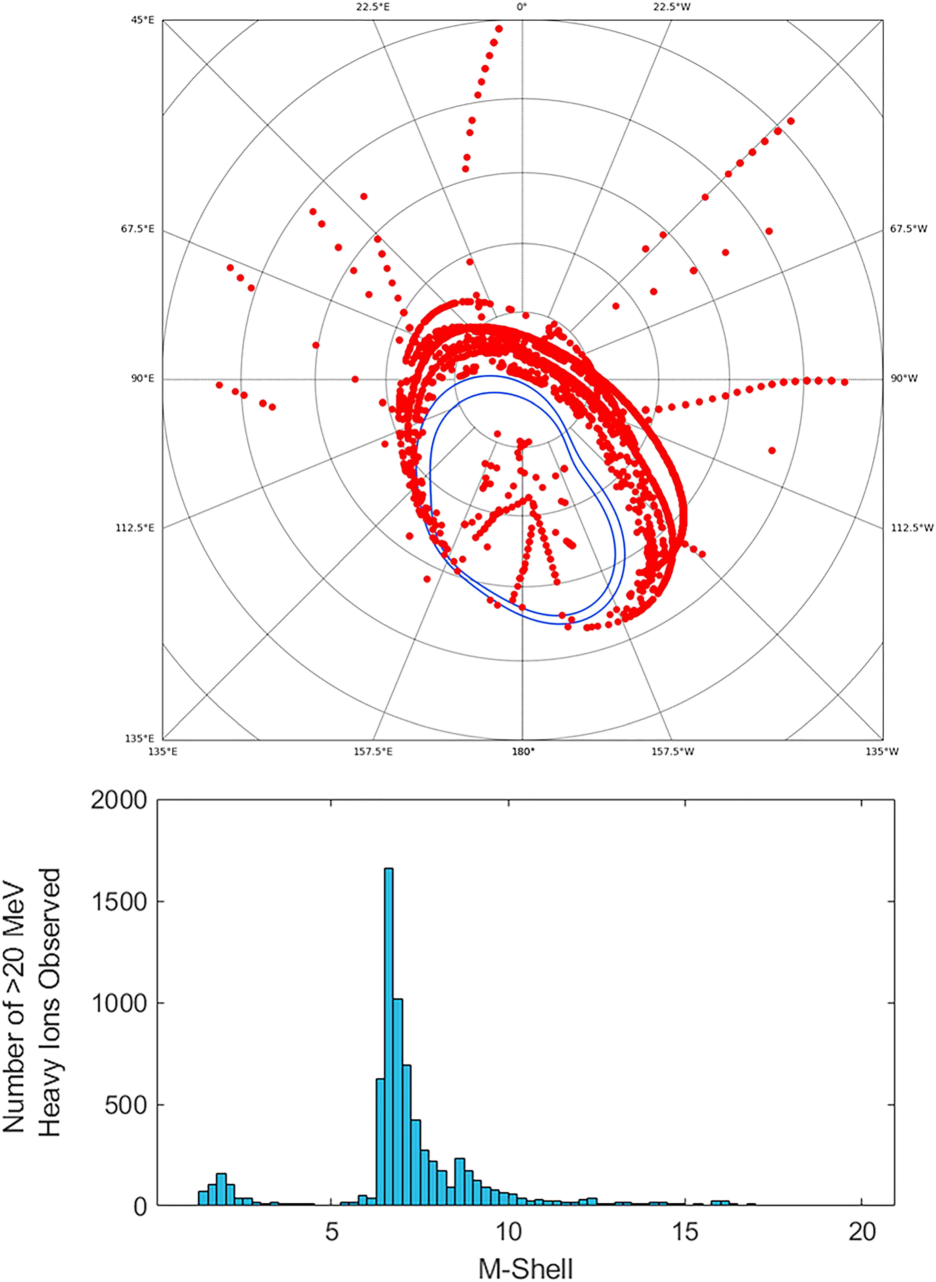
Locations on the Jovian north polar region where JEDI has measured oxygen and sulfur ions with energies greater than 20 MeV over the time period from 27 August 2016 to 30 October 2018. The red points are the mapped field line footprint locations of the Juno spacecraft during these measurements using the magnetic field model of Connerney et al. (2018). The majority of these measurements correspond to lower latitudes than the auroral oval (blue lines) thus possibly corresponding to trapped or precipitating particles. The lower histogram shows the distribution of these points versus M‐Shell in Rj confirming that the majority of these particles come from an M‐shell between 6 and 10.

Figure 4 shows the magnetic field line footprint locations of the JEDI measurements of oxygen and sulfur with energies above 20 MeV and up to 100 MeV. The footpoint locations are mapped onto the polar region of Jupiter using the magnetic field model of Connerney et al. (2018). The points plotted in Figure 4 are purely the locations that these energetic particles have been observed in the JEDI data, not a flux value. Due to the nature of the raw data products from Juno, it is not currently possible to retrieve fluxes from these points as they are collected fairly randomly. With additional study of the raw data collection methods, it may be possible to retrieve fluxes from this data; however, this is out of the scope of this study.

Interestingly, the majority of the observations of these heavy, energetic ions appear to correspond to the lower latitudes than the auroral oval and likely correspond to trapped or precipitating particles. The lower plot in Figure 4 shows a histogram of these locations versus Jupiter M‐Shell in R_J_, where “M‐Shell” in this case refers to the equatorial crossing distances for a magnetic field line using the magnetic field model of Connerney et al. (2018). There are a few significant populations within this plot; the first is that between the *M*‐Shells of 6 and 10 corresponding to the particles connected to the Io plasma torus.

## 4 Discussion and Conclusions

The very energetic ions measured by the JEDI instrument can coarsely be grouped into trapped and precipitating particles. The trapped particle group refers to very energetic ions that are quasi‐stably trapped in Jupiter's radiation belts. This group is nominally in the region from about *L* = 5 to *L* = 10 with higher concentrations at latitudes equatorward of the auroral oval. These particles can have very high bounce latitudes and so can be detected by Juno at different locations. At times they can also precipitate if their pitch angles are small enough, that is, within the atmospheric loss cone. The other group we report on here is the precipitating very energetic ions. These are particles that are likely associated with magnetic field lines that cross the equator at much greater distances than the planetary radiation belts. Since this group is precipitating, they likely interact with Jupiter's atmosphere and generate X‐rays. Because these observations do not contain information regarding the pitch angle of the particles, we cannot determine whether these particles are trapped or precipitating.

In addition to the majority of the points observed at lower latitudes than the auroral oval, there are several points observed poleward of the auroral oval. These particles could be sourced from the solar wind as it is known to contain significant populations of highly charged heavy ions that could precipitate into the Jovian atmosphere producing similar characteristic X‐rays (e.g., Dunn et al., 2017).

The most likely model of X‐ray generation by this mechanism is that low charge state precipitating heavy ions undergo stripping collisions as they pass through enough atmospheric material (e.g., Cravens et al., 1995). The orbital electron excitation transitions at high charge state have transition energies equivalent to X‐ray energies. The spatial distribution of Jovian X‐rays from the planet has been studied extensively. Dunn et al. (2017), for example, has found many X‐rays that are associated with field lines that map to the planetary magnetopause. A question then is how particles at those distances would reach the auroral region. Jupiter's magnetic field is so strong that the planetary loss cone at those distances is very small. The majority of the particles observed here are likely sourced from the radiation belts and have sufficient energy to produce X‐rays as they precipitate into the Jovian atmosphere.

In this paper, we have discussed how Juno JEDI obtains fluxes of very energetic ions using the raw data products, specifically the pulse width measurement. In contrast, the vast majority of particles detected by the TOFxE system rely on the pulse height, and these have been reported by Clark et al. (2017), Haggerty et al. (2017), and Mauk et al. (2017). The JEDI observations show heavy ions with energies up to and possibly greater than 100 MeV. These ions have masses that are consistent with oxygen and sulfur that likely originate from the volcanic plumes of Io and propagate through the Jovian system to then become trapped within Jupiter's magnetosphere or to precipitate into its atmosphere. These ions have sufficient energy that when they impact the Jovian upper atmosphere, they are rapidly stripped of their outer shell electrons, and subsequent electron capture from atmospheric neutrals into excited orbitals results in emission of characteristic X‐rays.

One of the outcomes of this study is a plan to modify the onboard JEDI tables to take advantage of the not fully realized capabilities reported here (this study, e.g., was restricted to the sparse raw event data set). In particular, a channel will be specifically tasked with characterizing the 20‐ to 100‐MeV heavy ions. It is only with such channel data that we can obtain good statistics throughout the orbit and properly characterize the intensity of the ions and compare with the theoretical expectations for generating the X‐rays.
